# Lymphedema and Therapeutic Lymphangiogenesis

**DOI:** 10.1155/2013/804675

**Published:** 2013-10-09

**Authors:** Yukihiro Saito, Hironori Nakagami, Yasufumi Kaneda, Ryuichi Morishita

**Affiliations:** ^1^Department of Vascular Surgery, Asahikawa Medical University, 2-1-1-1 Midorigaoka-Higashi, Asahikawa, Hokkaido 078-8510, Japan; ^2^Division of Vascular Medicine and Epigenetics, Department of Child Development, United Graduate School of Child Development, Osaka University, Kanazawa University, and Hamamatsu University School of Medicine, 2-1 Yamadaoka, Suita, Osaka 565-0871, Japan; ^3^Division of Gene Therapy Science, Graduate School of Medicine, Osaka University, 2-2 Yamadaoka, Suita, Osaka 565-0871, Japan; ^4^Department of Clinical Gene Therapy, Graduate School of Medicine, Osaka University, 2-2 Yamadaoka, Suita, Osaka 565-0871, Japan

## Abstract

Lymphedema is a disorder of the lymphatic vascular system characterized by impaired lymphatic return and swelling of the extremities. Lymphedema is divided into primary and secondary forms based on the underlying etiology. Despite substantial advances in both surgical and conservative techniques, therapeutic options for the management of lymphedema are limited. Although rarely lethal, lymphedema is a disfiguring and disabling condition with an associated decrease in the quality of life. The recent impressive expansion of knowledge on the molecular mechanisms governing lymphangiogenesis provides new possibilities for the treatment of lymphedema. This review highlights the lymphatic biology, the pathophysiology of lymphedema, and the therapeutic lymphangiogenesis using hepatocyte growth factor.

## 1. Introduction

 The lymphatic vascular system maintains tissue fluid homeostasis, plays a role in the afferent immune response, and carries proteins and large particulate matter away from the tissue spaces [1, 2]. Lymph stasis can accompany lymphatic anatomical or functional disorders as a result of both congenital and postnatal abnormalities. Because the lymphatic circulation provides the normal conduit for the return of interstitial fluid and protein to the blood circulation, abnormal lymph stasis creates an accumulation of protein and cellular metabolites in the extracellular space, resulting in an ensuing increase in tissue colloid osmotic pressure, water accumulation, and elevation of the interstitial hydraulic pressure. The lymphatic vascular system is a unidirectional transport system arising from blind ends. Fluid, cells, and macromolecules present in the interstitial space first enter blind-ended lymphatic capillaries. The lymphatic network permeates most organs in the body, as only the cornea, cartilage, epidermis, and central nervous system are devoid of lymphatic vessels. In addition to mammals, birds, fish, and amphibians also have a secondary lymphatic or lymphatic-like vascular system [3–6].

In addition to draining and transporting fluid, the lymphatic vascular system also plays an important role in the immune response by transporting leukocytes, antigens, and dendritic cells. Lymphatic vessels have many lymph nodes, which act as checkpoints for the immune response. The lymphatic system is also responsible for the absorption of dietary fats and fat-soluble vitamins from the digestive tract, in which specialized lymphatic capillaries (lacteals) in the intestinal villi absorb the lipid particles (chylomicrons) released by enterocytes.

 Anatomic or functional obstruction of the lymphatic system can result in the progressive accumulation of protein-rich fluid in the interstitial spaces (lymphedema) [7, 8]. Lymphedema is divided into primary and secondary forms based on the underlying etiology. Primary (hereditary) lymphedema results from genetic damage, whereas secondary (acquired) lymphedema is a consequence of lymphatic failure resulting from trauma, surgery, radiotherapy, or parasitic infection. Primary lymphedema is thought to occur in approximately 1–3 out of every 10,000 live births [9], irrespective of race or geographic area, and the female-male ratio is 3.5 : 1 [10]. The vast majority of lymphedema worldwide is secondary lymphedema. The most common cause of secondary lymphedema is filariasis. According to a 2013 report from the World Health Organization (http://www.who.int/mediacentre/factsheets/fs102/en/), lymphatic filariasis afflicts more than 25 million men with genital disease and more than 15 million people with lymphedema in Southeast Asia and African regions. In industrialized countries, cancer therapy is the leading cause of secondary lymphedema. Advanced malignancies frequently require radical surgery, including lymph node removal with or without radiotherapy, resulting in the destruction of the lymphatic vessel network. Approximately 30% of patients who have undergone breast cancer surgery develop lymphedema of the upper limb [11]. Even among patients treated with sentinel navigation surgery, approximately 6% develop lymphedema [11]. Furthermore, 10%–30% of patients with gynecological cancer develop lymphedema [12–14], as do approximately 15% of other lymphedema-related malignant tumor patients (16% for melanoma, 10% for genitourinary, 4% for head/neck tumors, and 30% for sarcoma) [15].

 Despite substantial advances in surgical and conservative techniques, therapeutic options for the management of lymphedema are limited [8, 16]. Although rarely lethal, lymphedema is a disfiguring and disabling condition which decreases the quality of life [17]. There is no cure for lymphedema at this time, but treatments to manage and reduce the swelling include physiotherapy, massage, and compression bandages, known as complex physical therapy [18]. 

## 2. Pathophysiology of Lymphedema 

**Figure 1:**
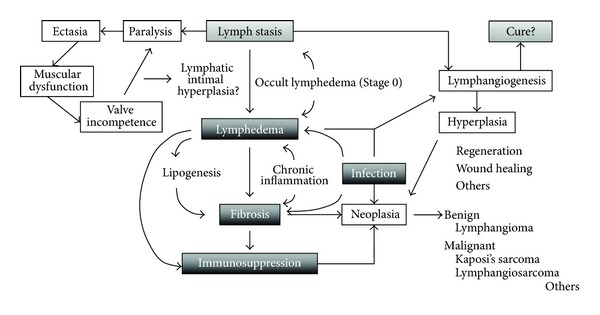
Schematic diagram of the pathophysiology of lymphedema.

 The pathophysiology of lymphedema is generally divided into two periods. During the first period, the pathological changes occur mainly only in the lymphatics and in the soft tissues lymphedema symptoms are not apparent (occult lymphedema = Stage 0). After this stage, the pathological changes occur in the soft tissue (fat, connective tissue, skin, etc.) of the limbs, resulting in the progressive swelling caused by systematic and combined pathologic factors. This clinical state is characterized not only by progressive swelling but also by fat and scar deposition, immunosuppression, a propensity for cellulitis, and microvascular proliferation (Figure 1).

### 2.1. Variable Period (Occult Lymphedema)

 Congenitally deficient or obstructed lymphatics promote lymph stasis, which is accompanied by deranged truncal contractility, progressive valve incompetence, destruction of contractile elements (lymphangioparalysis), and gradual ectasia of lymphatic collectors. After a variable period (occult lymphedema), a series of events is initiated, culminating in chronic lymphedema. Because of difficulties in diagnosis, the pathophysiology of occult lymphedema is almost unknown for most patients. 

### 2.2. Progress of Lymphedema and Exacerbation Factors

 When lymphedema is apparent via the pathological changes in the lymphatic system, some findings may be confirmed by images [18]: (1) obstruction of the lymphatic main route; (2) dermal back flow; (3) lack of lymph nodes; (4) existence of collateral lymphatic flow. As lymphedema worsens (Table 1), a decrease of swelling after limb elevation of the limb (Stage 1) will be not seen (Stage 2), and subsequently, the edema changes from pitting edema into nonpitting edema (late Stage 2). In many cases, the symptoms of lymphedema may be resistant to most of the therapies during this late Stage 2. Furthermore, edema is irreversible, and sclerosis of the skin and subcutaneous tissue (elephantiasis) may be remarkable (Stage 3) [18].

  These pathological changes in soft tissues are induced by fibrosis and metabolic disorder. At this time, chronic inflammation is recognized as the important mechanism, involving lymphocytes, monocyte/macrophages, and dendritic cells. As previously reported, these inflammatory cells produce many inflammatory cytokines related to fibrosis, such as CTGF, TGF-*β*, and PDGF. Cellular proliferation and migration of fibroblasts are upregulated [19].

Infection and adipogenesis are exacerbation factors of lymphedema. The propensity for recurrent soft-tissue infection is one of the most troublesome aspects of long-standing lymphedema. Accumulated fluid and proteins provide a good substrate for bacterial growth. Lymphatic dysfunction impairs the local immune response, which plays a permissive role in the propagation of bacterial and fungal invasion. Furthermore, once established, soft tissue infection exacerbates the existing lymphatic dysfunction, sometimes irreversibly.

Although the connection between the lymphatic system and fat absorption/deposition has been recognized by clinicians for well over a hundred years, the subject received relatively little interest until recent publications raised theories regarding the mechanism of this association. It has long been recognized that a lymphedematous limb accumulates fat at an increased rate when compared to the rest of the body and that, conversely, when weight loss is undertaken, the lymphedematous limb loses fat at a slower rate than the rest of the body. The underlying cause of these observations is not well understood. A recent publication studying Prox1 haploinsufficient mice proposed that the lymph itself is stimulatory to fat cells [20]. According to recent reports, adipocytes recruit monocyte/macrophages via activation of NF*κ*B and TNF-*α* [21]. Furthermore, it was reported that adipocytokines participate in fat absorption [22].

### 2.3. Primary Lymphedema Related Genes

Three genes were confirmed as the cause of lymphedema [23, 24]: (1) VEGFR-3 (familial Milroy lymphedema); (2) FOXC2 (lymphedema-distichiasis syndrome); (3) SOX18 (hypotrichosis-lymphedema-telangiectasia). In addition to these genes, the following genetic changes are associated with lymphedema: Aagenaes syndrome (chromosome 15), Noonan's syndrome (chromosome 12), trisomy disorders (chromosome 13, 18, 21, and 22), Klinefelter's syndrome (XXY), Turner's syndrome (XO), and chromosomal abnormalities of additions at 11p and deletions at 11q and 13q.Klippel-Trenaunay syndrome is recognized as a nonhereditary disease. 

### 2.4. Malignancy in Patients with Lymphedema

 Although lymphedema is recognized to generally not affect prognosis of mortality, in rare cases, chronic lymphedema may be complicated by the development of malignant tumors. One of these malignancies is lymphangiosarcoma (Stewart-Treves syndrome) [25]. The incidence rate of this syndrome in patients with lymphedema 5 years after breast cancer surgery is 0.07–0.45%, according to previous reports [26]. Other malignant tumors that appear with increased frequency in lymphedematous limbs include Kaposi's sarcoma, squamous cell carcinoma, malignant lymphoma, and melanoma. These malignancies frequently result in limb loss or even death. The mechanism of development of malignancy is unclear, but because these malignancies occur in the chronic lymphedema patients of any cause, the lymphedema state is thought to affect the development of these malignancies.

## 3. Therapeutic Lymphangiogenesis

### 3.1. Development on Lymphangiogenesis

Many reports regarding the molecular biology of lymphatics and lymphangiogenesis were published during the 1990s, in accordance with progress in the field of vascular biology. These developments were supported by the identification of lymphatic specific markers, such as LYVE-1 and Prox1, and subsequent improved ability to easily observe the lymphatics or lymphatic endothelial cells (LEC). 

 Recent studies suggest that lymphangiogenesis can be stimulated by various cytokines. For example, vascular endothelial growth factors (VEGF)-C and -D promote lymphangiogenesis by activating the VEGF receptor-3 (VEGFR-3), which is expressed on lymphatic endothelial cells (LEC) [27]. As further evidence, VEGF-C-deficient mice fail to develop a functional lymphatic system [28], transgenic expression of soluble VEGFR-3 results in pronounced lymphedema [29], and gene transfer of VEGF-C effectively reduces lymphedema in an animal model [30]. Another study reported that angiopoietin-1 also promotes lymphatic vessel formation through Tie2 [31] and that fibroblast growth factor 2 stimulates the growth of lymphatic vessels [32].

### 3.2. HGF Gene Therapy for Lymphedema

Hepatocyte growth factor (HGF) was originally identified as a potent mitogen for hepatocytes, and HGF is currently recognized as a mesenchyme-derived pleiotropic factor that regulates growth, motility, and morphogenesis of various types of cells [33–37]. Furthermore, HGF plasmid DNA is utilized for gene therapies targeting the heart [38], vascular system [39], brain [40], and lung [41]. HGF activates its tyrosine kinase receptor, c-Met [33, 42], and various c-Met-linked intracellular signaling pathways, such as the Ras-mitogen-activated protein kinase cascade (MAPK) or the phosphatidylinositol-3-OH kinase (PI3 K)-Akt cascade [43, 44].

**Figure 2:**
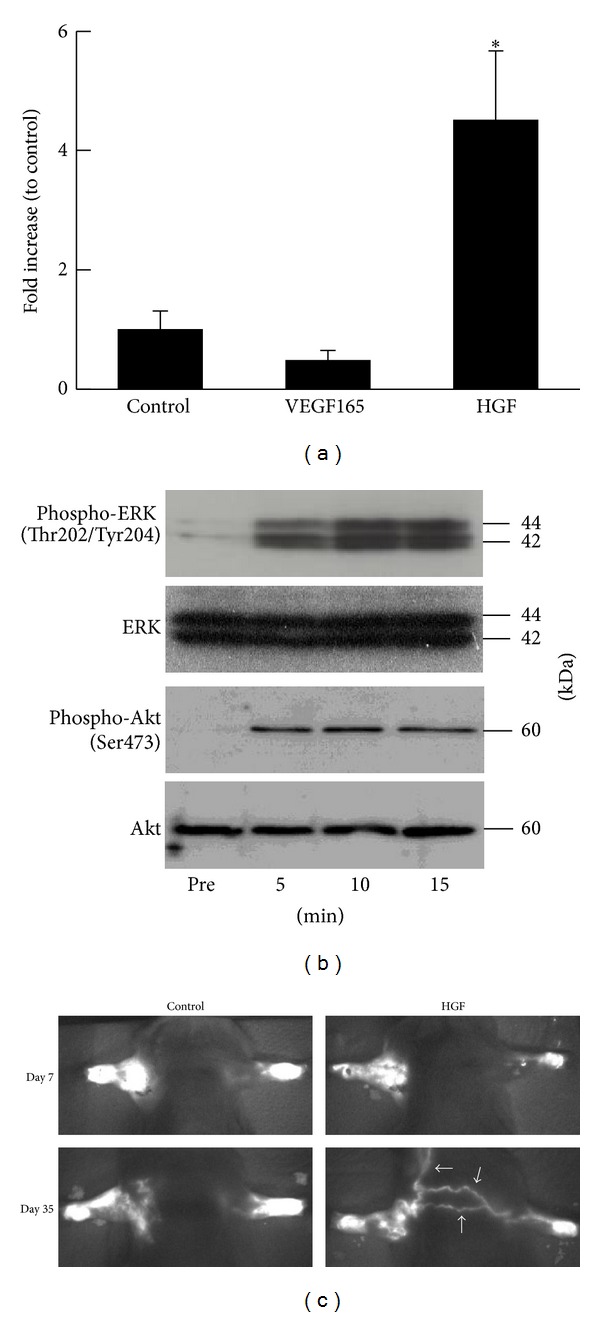
HGF lymphangiogenesis and gene therapy for lymphedema. (a) Effect of HGF plasmid on c-fos promoter activity in LEC. *n* = 4, *P < 0.05 versus control, VEGF165. (b) Typical western blot of ERK or Akt and phosphorylated ERK or Akt in cLEC before and 5, 10, and 15 minutes after treatment with human recombinant HGF (50 ng/mL). (c) Representative pictures of the fluorescent lymphography using PDE at day 7 and day 35 after surgery. “HGF” indicates human HGF plasmid (200 *μ*g/0.1 mL) and “control” indicates GFP plasmid injection (200 *μ*g/0.1 mL).

We have previously investigated and reported the lymphangiogenic potency of HGF [45]. Canine lymphatic endothelial cells (cLEC) express c-Met as demonstrated by immunofluorescent staining, suggesting that cLEC are responsive to HGF. Indeed, the treatment of cLEC with HGF resulted in a dose-dependent increase in cellular proliferation and migration (Figure 2(a)). Furthermore, the extracellular signal-regulated protein kinase (ERK) or Akt was phosphorylated 5–15 minutes after the addition of HGF to cLEC, whereas total ERK or Akt protein levels were not altered by treatment with recombinant HGF (Figure 2(b)).

In accordance with *in vitro *data, we confirm the effect of HGF gene transfer for *in vivo* models. Using the mouse upper limb lymphedema model in a simulation of breast cancer related lymphedema, the operated arm volume began to increase 1 day after the operation and was stable at 7 days after operation in all animal groups. The arm volume of the HGF injected group was significantly decreased on postoperative day 7, compared with the arm volumes of the control group. This significant difference between the HGF injected group and the control group continued to postoperative day 35. Of note, new extra-anatomical lymphatic flow was observed only in the HGF injected group, as detected by the fluorescent lymphography system (PDE; Hamamatsu Photonics K.K. Hamamatsu, Japan, Figure 2(c)). We hypothesized that these lymphatic flows are induced by HGF lymphangiogenic potency.

In view of these results, we hypothesized that HGF gene therapy would stimulate the growth of the lymphatic vascular system and alter the course of lymphedema. In terms of HGF gene therapy, the safety of the use of HGF plasmid DNA in patients with critical limb ischemia has been investigated in a prospective open-labeled clinical trial [46]. There were no signs of systemic or local inflammatory reactions and no development of tumors or progression of diabetic retinopathy in this population. Of note, no edema was observed in this trial, in contrast to the transient lower-extremity edema that was reported after the use of clinical gene therapy using the VEGF-A gene [47]. We are currently preparing to start a clinical trial involving lymphedema patients and expect to observe successful therapeutic lymphangiogenesis management by HGF gene therapy.

## 4. Conclusion

 The lymphatic vessels have three specific functions for maintenance of homeostasis: (1) drainage of tissue fluid; (2) immunosurveillance; and (3) the uptake of dietary fats. Furthermore, they play an important role in the pathogenesis of several diseases, including cancer, lymphedema, and various inflammatory conditions. Consequently, administration of lymphatic growth factors or related molecules provides the potential to target lymphatic vessels in human disease. In particular, therapeutic lymphangiogenesis is a promising gene therapy strategy for the treatment of lymphedema. 

## Figures and Tables

**Table 1:** Staging according to the “consensus document” of the International Society of Lymphology.

Clinical stage	Evidence
0	Subclinical with possible clinical evolution
I	Edema regressing with treatments with positive pitting test
II	Edema partially regressing with treatments with negative pitting test
III	Elephantiasis with cutaneous complications and recurrent infections
